# Safety and feasibility of third-party multipotent adult progenitor cells for immunomodulation therapy after liver transplantation--a phase I study (MISOT-I)

**DOI:** 10.1186/1479-5876-9-124

**Published:** 2011-07-28

**Authors:** Felix C Popp, Barbara Fillenberg, Elke Eggenhofer, Philipp Renner, Johannes Dillmann, Volker Benseler, Andreas A Schnitzbauer, James Hutchinson, Robert Deans, Deborah Ladenheim, Cheryl A Graveen, Florian Zeman, Michael Koller, Martin J Hoogduijn, Edward K Geissler, Hans J Schlitt, Marc H Dahlke

**Affiliations:** 1 Department of Surgery, University Medical Center Regensburg, Regensburg, Germany; 2 Athersys Inc., Cleveland, Ohio, USA; 3 Center for Clinical Studies, University Medical Center Regensburg, Regensburg, Germany; 4 Department of Internal Medicine, Erasmus Medical Center, Rotterdam, The Netherlands

## Abstract

**Background:**

Liver transplantation is the definitive treatment for many end-stage liver diseases. However, the life-long immunosuppression needed to prevent graft rejection causes clinically significant side effects. Cellular immunomodulatory therapies may allow the dose of immunosuppressive drugs to be reduced. In the current protocol, we propose to complement immunosuppressive pharmacotherapy with third-party multipotent adult progenitor cells (MAPCs), a culture-selected population of adult adherent stem cells derived from bone marrow that has been shown to display potent immunomodulatory and regenerative properties. In animal models, MAPCs reduce the need for pharmacological immunosuppression after experimental solid organ transplantation and regenerate damaged organs.

**Methods:**

Patients enrolled in this phase I, single-arm, single-center safety and feasibility study (n = 3-24) will receive 2 doses of third-party MAPCs after liver transplantation, on days 1 and 3, in addition to a calcineurin-inhibitor-free "bottom-up" immunosuppressive regimen with basiliximab, mycophenolic acid, and steroids. The study objective is to evaluate the safety and clinical feasibility of MAPC administration in this patient cohort. The primary endpoint of the study is safety, assessed by standardized dose-limiting toxicity events. One secondary endpoint is the time until first biopsy-proven acute rejection, in order to collect first evidence of efficacy. Dose escalation (150, 300, 450, and 600 million MAPCs) will be done according to a 3 + 3 classical escalation design (4 groups of 3-6 patients each).

**Discussion:**

If MAPCs are safe for patients undergoing liver transplantation in this study, a phase II/III trial will be conducted to assess their clinical efficacy.

## Background

### Liver Transplantation

Liver transplantation remains the only definitive treatment for a number of diseases, including end-stage chronic liver disease, acute liver failure, or limited hepatic neoplasms, with patient and graft survival rates exceeding 75% after five years [1,2]. However, liver transplantation is burdened by the need for life-long immunosuppression in order to prevent graft rejection. All drugs currently used for immunosuppression cause significant clinical side effects. Besides their well-known intrinsic toxicities (e.g., neurotoxicity of tacrolimus and renal toxicity of ciclosporin [3-5]), they also increase the risk for cancer and opportunistic infections [6-11]. The long-term overall success of liver transplantation is frequently determined by complications related to immunosuppressive drug therapy. Yet, immunosuppressants are indispensable to maintain graft function and to cover aberrations in immune reactions that may result in rejection of the transplanted organ.

Growing numbers of patients in need of a liver graft are faced with a continuous shortage of donor organs. In the Eurotransplant area, for instance, only 1631 transplant livers were available for 2641 patients on the waiting list in 2009 [12]. To overcome this shortage, criteria for the acceptance of donors have been liberalized, e.g., in terms of prolonged ischemia time, increased donor age, or the presence of clinically significant donor liver steatosis. While increasing the donor pool, these "marginal" organs are also associated with higher incidences of primary graft dysfunction and major complications [13-15]. Here, we propose a novel protocol involving treatment of liver transplant recipients with multipotent adult progenitor cells (MAPCs) with the goal of reducing the dose of immunosuppressive drugs and of supporting liver regeneration in marginal grafts.

### Multipotent adult progenitor cells

MAPCs belong to the family of mesenchymal stem cells (MSCs) and are cultured from bone marrow aspirates [16-18]. The clinical-grade MAPC product (MultiStem^®^, Athersys Inc., Cleveland, Ohio, USA) to be used in this study is isolated from a single bone marrow aspirate and cultured with heat inactivated fetal bovine serum (FBS) and growth factors EGF and PDGF. Cells display a linear expansion rate to 65 population doublings or greater before senescence. Doubling times average 20 hours during expansion. Cells are used after 30 population doublings and tested by flow cytometry, in vitro immunomodulatory assays and cytogenetics. Moreover, extensive safety testing in immunodeficient animal models is performed [19-21].

MAPCs share immunosuppressive functions with MSCs [16], they have been shown to suppress T-cell proliferation in vitro and ameliorate graft-versus-host disease (GvHD) in small animal models [22]. First clinical trials with MAPCs have already been initiated to treat GvHD and Crohn's disease [21]. Moreover, MAPCs have regenerative properties, contributing to vascular regeneration in models of limb ischemia [23], improving cardiac function after myocardial infarction [24], and contributing to the regeneration of injured livers through their ability to differentiate into hepatocyte-like cells [25].

MSCs and MAPCs have been successfully applied in preclinical heart transplantation models in combination with various immunosuppressants [26-29]. Our group has demonstrated that MSCs and MAPCs induce long-term graft acceptance when applied together with mycophenolic acid [26,27]. In contrast, calcineurin inhibitors (CNIs) have been shown to abrogate the immunosuppressive effect of MSC therapy in this and other animal models [30]. The current study protocol therefore calls for a CNI-free, "bottom-up" immunosuppressive regimen combined with the MAPC infusions.

### "Bottom-up" immunosuppression

Current standard clinical protocols for post-transplant immunosuppression vary between institutions, continents and indications. However, most induction therapies include corticosteroids that are subsequently tapered over the first months. CNIs, such as ciclosporin A or tacrolimus, are the mainstay of immunosuppression, sometimes in combination with mycophenolic acid (MPA). Further treatment options are also available, like e.g. thymoglobulin. In addition, anti-CD25 monoclonal antibodies can be used to block activated T cells in the first week after the operation [31]. Because standard immunosuppressive treatment is often reliant on CNI-based regimens, which can cause among other things renal impairment, hypertension, and hyperglycemia [32-35], efforts have been made to reduce CNI exposure for liver transplant recipients [36]. Indeed, a proportion of patients can achieve graft acceptance without CNIs, while acute rejection episodes in the remaining patients can be treated with high-dose steroids and intensification of the baseline immunosuppressive regimen, without graft loss.

"Bottom-up" immunosuppression, then, refers to a CNI-free induction protocol consisting of steroids, mycophenolic acid and basiliximab. CNIs are introduced only when needed, e.g. in case of biopsy-proven acute rejection. This approach is feasible in liver transplantation, because of its lower immunogenicity in comparison to other types of organ transplants and because of the low risk of graft loss or permanent graft damage by acute rejection episodes. The "bottom-up" regimen has already been applied successfully in clinical studies [37,38] and is particularly valuable for high-MELD (Model for End-stage Liver Disease) patients with increased risk of infections or renal dysfunction. In view of the synergistic interplay of MSCs with mycophenolic acid, and because CNIs have been shown to abolish the beneficial effect of MSCs in animal models, this study will use "bottom-up" immunosuppression in combination with MAPCs. We hypothesize that MAPC infusions will help to significantly delay the introduction of CNIs or allow to avoid them altogether.

## Methods & Design

### Objectives and Endpoints

The primary objective of this study is to assess the safety of MAPC infusions in patients undergoing liver transplantation. The secondary objective is to provide preliminary evidence regarding the study product's efficacy by analyzing the time to first biopsy-proven acute rejection up to day 90. Furthermore the incidence of malignancies or any other unexpected side effects until day 365 will be investigated. After closing this study, all participants will be enrolled in a follow-up protocol that assesses long-term safety of MAPCs over an additional 6 years. This two-step follow-up approach has been designed in close collaboration with the responsible regulatory authorities. Immunomonitoring will be performed on blood samples from all participating patients to assess the anti-donor immune response, the composition of circulating T cell subpopulations, the anti-donor antibody response and to identify a putative biomarker signature that is associated with transplant tolerance.

### Study Design

**Figure 1:**
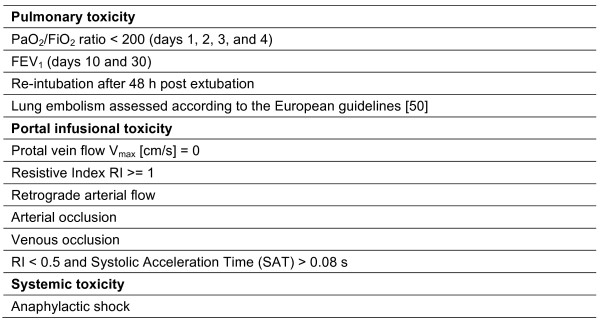
**Dose-limiting toxicity (DLT) events.**Clinical events of toxicity related to MAPC infusions. If more than one DLT event occurs in a dose cohort, the study will be stopped.

This is a phase I, single-arm, single-center safety and feasibility study based on a classical 3 + 3 dose escalation design. Safety of MAPC infusions is assessed by the occurrence of a dose-limiting toxicity (DLT) event (Figure 1) within 30 days after administration of the first MAPC dose. Because the focus in this study is on safety, a conservative dose escalation scheme rather than an accelerated titration design was chosen. The starting dose of 2 × 150 million MAPCs (MultiStem^®^) per patient has already been administered to patients for various indications, with no side effects observed so far. This dose corresponds to doses that have been shown to prolong graft survival in animal models. The maximum dose of 2 × 600 million MAPCs is still at least 50% lower than the maximum-tolerated dose in laboratory animals and similar to MSC doses already injected into patients [39].

**Figure 2:**
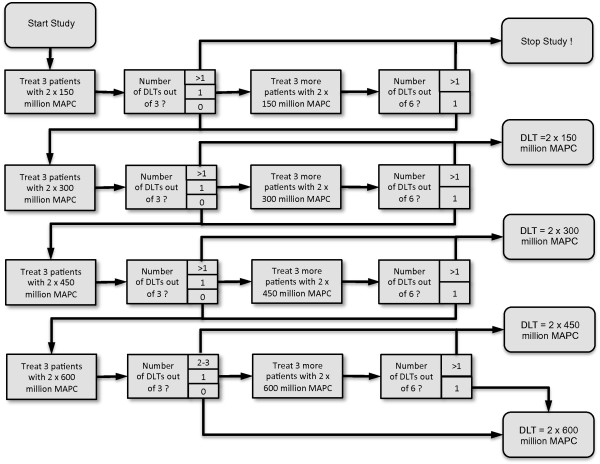
**Dose escalation design**. Three patients will be treated with the starting dose. If no DLT occurs, the next cohort will be treated with the next MAPC dose level. If one DLT occurs in a cohort, a second cohort of 3 patients will be treated with the same MAPC dose level. The study will be stopped if more than one DLT event is recorded after enrolling at most 6 patients.

Each patient will receive 2 doses of MAPCs. The first dose will be administered during liver transplantation directly into the portal vein after graft reperfusion. As the study begins with liver transplantation this day is defined as day 1 (in contrast to most preclinical investigation that defines the day of the transplant as "day 0"). The second dose will be administered intravenously on day 3 in the intensive care unit. Three patients will be treated with the starting dose of 2 × 150 million third-party MAPCs. If no DLT is observed in any of the 3 patients of this cohort, the second cohort of 3 patients will be treated with 2 × 300 million MAPCs, continuing with the third cohort with 2 × 450 million MAPCs and the fourth cohort with a final dose of 2 × 600 million MAPCs. The dose escalation design is illustrated in Figure 2.

Should one patient experience a DLT after 3 patients have been enrolled in any cohort, another 3 patients will be enrolled in the same dose group after consultation with the data safety monitoring board. If no further toxicity occurs, the next 3 patients will be enrolled at the next dose level. If a total of 2 or more patients experience a DLT, either after 3 or 6 patients have been enrolled, the study will be closed and the dose of the previous cohort will be considered the maximum-tolerated dose.

If no toxicities occur at all, the maximum dose administered in the study, i.e., 2 × 600 million MAPCs per patient, will be considered the maximum-tolerated dose and the study will be closed. Using the dose escalation scheme described above, between 3 and 24 patients will be enrolled in this study, with 12 patients being the optimal scenario. The study protocol was designed according to the declaration of Helsinki and approved by the local ethics committee.

### Trial Population

**Figure 3:**
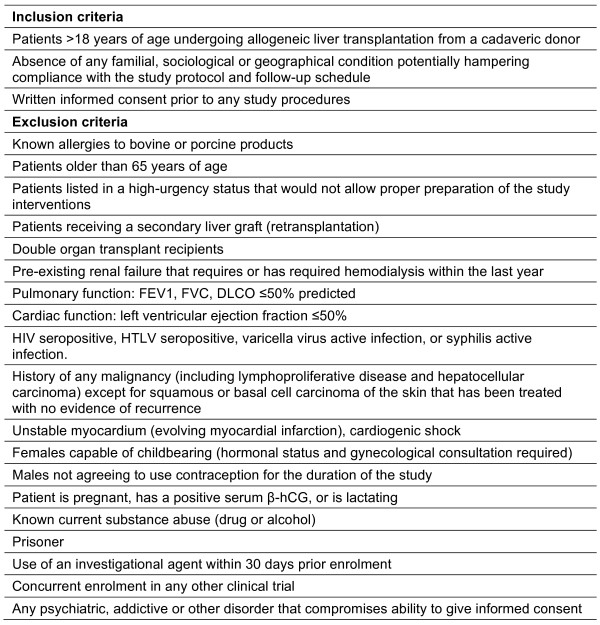
Inclusion and exclusion criteria of the study.

Patients of both genders and any ethnic origin aged 18 years or older will be screened at the Department of Surgery, University Hospital Regensburg, and enrolled into the study if they meet the eligibility criteria given in Figure 3. All suitable patients will be informed about the study during a regular outpatient visit and asked for their willingness to participate. Specific study related risks such as the possible transmission of xenopathogens following cell culture with bovine serum will be explained. At our institution annually 70-80 patients are placed on the European Liver Transplant Waiting List. Therefore, to enroll 3-24 patients for the study, a recruitment period of 12 months is anticipated.

### Interventions

#### Pre- and Intraoperative Data

Patients enrolled in this study will not need to undergo additional screening visits or clinical investigations in addition to standard pre-transplant work-up. Standard-of-care examinations for patients on the Liver Transplant Waiting List will be performed, including baseline clinical data (demographics, medical history, current medication), physical examination, laboratory examinations, infection screening, urinalysis, electrocardiogram, echocardiography, chest X-ray, triple-phase abdominal computed tomography with intravenous and oral contrast, pulmonary function tests, and arterial blood gas analysis. Intraoperative data (warm and cold ischemia time, blood loss, requirement for blood products, incision-to-suture time) and donor data (age, serum sodium and gamma-GT levels, body mass index, infection status, cause of death, time on intensive care unit) will also be documented.

#### Treatment Regimen

Immunosuppression will be tailored to the individual needs of each patient in a "bottom-up" approach. The immunosuppressive protocol used in this study is already being applied at our center in patients with an expected low risk for rejection (MELD score > 25, particularly with preoperative renal dysfunction). Prior to liver reperfusion, 500 mg prednisolone will be administered intravenously. The cell product stored in liquid nitrogen at our hospital blood bank will be thawed by a qualified person and prepared for application. After liver reperfusion, the transplant surgeon will infuse the first MAPC dose from the freshly thawed cryobag directly into the portal vein using a small catheter.

On days 1 and 5, 20 mg of basiliximab will be administered for induction therapy as one key element of the institution's immunosuppressive regimen. There is a growing body of evidence indicating that basiliximab can impair the development of transplant tolerance by preventing the development of regulatory T cells [40-43]. Since we anticipate that omitting basiliximab will not influence MAPC toxicity, we have chosen to retain basiliximab yet to focus solely on safety in this study. More preclinical data is then needed to establish a causal relationship between basiliximab and putative MAPC effects. If it turns out that MAPCs depend on intact interleukin-2 signaling, the application of basiliximab in a subsequent efficacy study has to be critically discussed.

**Figure 4:**
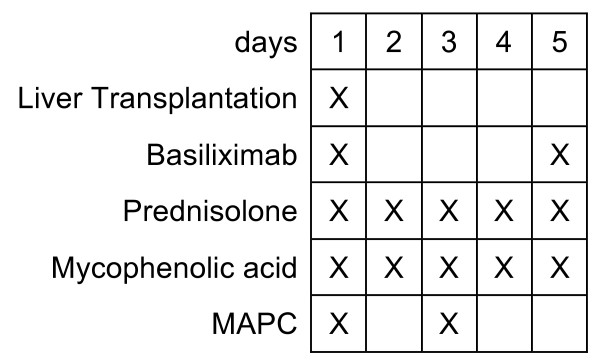
**Immunosuppressive treatment regimen**. Basiliximab will be applied on days 1 and 5 after transplantation; 2 g mycophenolic acid (MPA) will be applied per day given as a split dose. Steroids will be started on postoperative day 1 and tapered by month 6 after liver transplantation, MAPC infusions will be administered into the portal vein during transplantation and later intravenously on day 3.

Maintenance immunosuppression will be conducted with 2 g/d mycophenolic acid (MPA) given as a split dose twice daily. Steroids at a dose of 1 mg/kg body weight will be commenced on day 1 and tapered successively. On day 3, the second MAPC dose will be administered intravenously in the intensive care unit. All patients will be monitored in a fully equipped tertiary intensive care unit before and for at least 48 hours after the cell infusion (see Figure 4).

#### Follow-up

Thirteen follow-up visits will be performed during the first 30 days after transplantation. Blood samples will be collected, clinical examinations performed, and adverse events recorded as detailed in Table 1. Dose-limiting toxicity (DLT) assessments will be performed on days 1, 2, 3, 4, 10, and 30. Per protocol, biopsies will be performed during liver transplantation and on days 4 and 10, with additional biopsies obtained whenever clinically necessary. Four additional outpatient visits are planned to further evaluate the study patients (including screening for malignancies) until day 365 (Table 1). Additional blood samples will be obtained to investigate surrogate markers of the patient's immune response status. This translational immunomonitoring will be performed on days 1, 3, 10, and 30, including mixed lymphocyte reactions to evaluate anti-donor reactivity, flow cytometry to describe the recipients' leucocyte repertoire, serum analysis to screen for anti-donor antibodies and cytokines. Moreover, we will analyze peripheral blood samples for genes that have recently been associated with tolerance in liver and kidney transplantation such as CKLRF1, CLIC3 and TOAG-1 [44-48]. Using specific donor characteristic (e.g. differences in gender or MHC haplotypes) circulating MAPC will be tracked in blood samples by rtPCR. Further labeling of transfused MAPC is not planned at this stage for safety reasons. We expect MAPC to be cleared quickly from the recipient because they have been susceptible to NK-cell lysis and were detected only transiently in most animal experiments [49].

**Table 1:** Assessment schedule.

Study Phase	LTx		EOT	Post-Treatment Phase						Follow-Up			
**Visit no**.	**1**	**2**	**3**	**4**	**5-9**	**10**	**11**	**12**	**13**	**14**	**15**	**16**	**17**

Study days	1	2	3	4	5-9	10	14	20	30 ± 10 d	90 ± 30 d	180 ± 30 d	270 ± 30 d	365 ± 30 d

MAPC treatment	X		X										

DLT assessment	X	X	X	X		X			X				

Laboratory	X	X	X	X	X	X	X	X	X	X	X	X	X

Doppler ultrasound	X	X	X	X		X			X	X			X

Liver biopsy	X			X		X							

Immunomonitoring	X		X			X			X				X

### Dose-Limiting Toxicity

To assess the safety of MAPC infusions, we have defined putative toxicity events anticipated to be specific for stem cell-based therapy in liver transplantation. This dose-limiting toxicity (DLT), which covers specific events that model significant toxicity likely caused by MAPC infusions, is designed as a 'high-barrier score' that aims to detect toxicities of the highest clinical significance that will halt the further development of this therapy option.

The most important consideration is that MAPCs might pool in the first capillary bed after injection and cause micro- or macroembolism. To monitor for potential embolus formation, we have specified diagnostic procedures to examine the liver and lung after intraportal and intravenous injection, respectively. Toxicity related to intraportal infusion will be assessed by Doppler ultrasound determining the maximum portal blood flow, the resistive index (RI) of the hepatic artery, and the presence of any vascular occlusion or changes in the flow patterns. We will monitor lung toxicity by assessing the necessity of reintubation and the occurrence of pulmonary emboli according to published European guidelines after intravenous cell infusion [50]; moreover, the PaO_2_/FiO_2 _ratio [51] will be tightly monitored to detect lung damage. Because MAPCs are derived from a third-party donor and were cultured with bovine serum and recombinant growth factors, MAPC infusion may cause anaphylactic reactions or shock, and systemic toxicity will therefore also be assessed (Figure 1). Three more patients will be enrolled into a dose cohort if one DLT event occurs. The study will be stopped if more than one DLT event occurs after enrolling 6 patients or if the data safety monitoring committee recommends to do so. The feasibility and validity of the DLT events have been validated in 200 retrospectively analyzed patients having received liver grafts without experimental cellular therapy (unpublished data).

### Data safety monitoring committee

An independent data safety monitoring committee will be installed to monitor the study progress. The committee will include basic scientists and clinicians not otherwise involved in the trail. Members of this group will review the clinical and investigational data to ensure that participants are not exposed to undue risk. The data safety monitoring committee will review the data up to day 30 for each dosing cohort and will then give written recommendation on whether or not to continue the study. Members of the committee will also recommend on whether the next dosing cohort should start enrolment or whether the current cohort should be expanded. The data safety monitoring committee can recommend stoppage of the study for reasons of patient safety at any time. Whenever adverse events occur, the principal investigator and the study team will communicate those to the data safety monitoring committee in due time. If an adverse event is serious (SAE) or unexpected (SUSAR), the responsible authorities will be informed. About 10 SAEs might be expected in each liver transplant recipient transplanted with high MELD score during the first 30 days.

### Risk-Benefit Assessment

Although pharmacological immunosuppression has continuously evolved over the last three decades, it is still associated with a significant intrinsic risk. Side effects include opportunistic (mainly biliary) infections in the short-term and drug-specific side effects or malignancies in the intermediate and long-term [9,52]. Thus, even in this era of established immunosuppressive pharmacotherapy, there is still significant room for improvement of current immunosuppressive protocols. Moreover, long-term survival of liver transplant recipients has not improved over the past decade, suggesting novel strategies are needed to extend life after transplantation.

Adherent, non-hematopoietic bone marrow stem cells, including MAPCs and MSCs, have been shown to beneficially modulate the anti-donor immune response in organ transplantation and to promote tissue regeneration in vitro and in vivo [26-29,53]. The first promising experiences using MAPCs in patients with autoimmune disorders, such as inflammatory bowel disease or GvHD, have been reported. Other conditions, especially those requiring regenerative support, such as critical limb ischemia or myocardial infarction, have also successfully been treated with MAPCs in animal models [23,24]. It is therefore clinically promising to test the application of MAPCs in a phase I study after allogeneic liver transplantation. The risk of applying MAPCs to this patient population is unknown. However, so far no significant side effects of MAPC infusions have been observed in either animal disease models or in phase I and II clinical studies in humans. Thus, we believe that the potential benefit of administering MAPCs to patients after allogeneic liver transplantation is significant and that the associated risks of the cell infusions are low and tolerable. In summary, the benefits of MAPC infusions promise to outweigh the risks.

## Discussion

Standard pharmacological immunosuppression can achieve good survival of patients and liver grafts [1,2,12]. This success of interdisciplinary transplant medicine has made liver transplantation a standard-of-care clinical therapy for end-stage liver disease. Long-term side effects of organ transplantation with chronic immunosuppressive therapy, however, are clinically significant and limit the overall success of the procedure [3-11]. Therefore, the objective of this study is to implement cellular immunomodulation therapy as an adjunct to standard pharmacological immunosuppression. The ultimate goal of this approach is to significantly reduce drug-based immunosuppression and achieve a state of long-term transplant acceptance completely without immunosuppression for some recipients. To apply MAPCs in the clinic, we think that the calcineurin inhibitor-free "bottom-up" immunosuppression regime is essential because animal data suggest a synergistic effect of MSCs with mycophenolic acid and an antagonistic effect of MSCs with cyclosporine [26,27,30,54]. Therefore, in our view the liver is the most promising organ to establish a MAPC-based therapy because it is the only organ that can be transplanted without using calcineurin inhibitors routinely. In case acute rejection occurs despite MAPC treatment, this can be treated with a low risk of graft loss or permanent graft damage justifying the attempt to reduce drug-based immunosuppression with MAPCs.

The main focus of this phase I study is on safety and feasibility of infusing a population of MAPCs with suspected immunomodulative and regenerative features. Therefore, the primary endpoint is the occurrence of dose-limiting toxicity events. To explore for immunological efficacy, secondary endpoints include the time until first biopsy-proven acute rejection (up to day 90). From another view, one of the secondary endpoints is to look for evidence of malignant transformation of the infused cells that would severely limit their further use. Long-term persistence of MAPC might be associated with a higher potential of malignant transformation and recipient-anti-donor-sensitization. Therefore we will attempt to track circulating MAPCs in peripheral blood samples by rtPCR. Further labeling of the transfused cells cannot be justified in this phase I trial for reasons of patient safety.

The hypothesis is that MAPCs can prevent acute rejection episodes in the early post-transplant phase by interaction with recipient lymphocytes. We anticipate shifting the immune response towards a state of permanent graft acceptance that makes the escalation of pharmacological immunosuppression unnecessary. Moreover, we expect MAPCs to ameliorate ischemia/reperfusion damage to the graft, thereby avoiding late complications, such as hepatorenal syndrome and bile duct ischemia. The regenerative abilities of MAPCs could also reduce the occurrences of primary graft dysfunction and accelerate normalization of liver synthesis function especially in marginal liver grafts.

In summary, the expected clinical efficacy of MAPC infusions as an adjunct to established immunosuppressive pharmacotherapy is substantial and the potential benefits outweigh the expected risks. MAPCs have already been administered in about 50 patients with no specific severe side effects reported [55]. MSCs, which can be considered similar to MAPCs in terms of their safety profile, have been administered in over 200 patients with no reported malignancies or severe side effects [56]. If the lack of dose-limiting toxicities can be confirmed in the present study, we intend to conduct a second, larger study to assess the immunomodulatory and regenerative efficacy of MAPC infusion in liver transplantation. A positive outcome from MAPC therapy trials in terms of reducing the need for pharmacological immunosuppression would represent a major advancement for liver transplant recipients.

## Competing interests

MHD receives funding from Athersys and Novartis to conduct the study.

## Authors' contributions

MHD designed the study with EE, BF, PR, FCP, and HJS. BF developed essential study documents. EKG, PS, and PP supported the design of the study with their knowledge and experience. MHD is the principal investigator of the study and the sponsor's representative. All authors have read and approved the final manuscript.
